# A differential loss of nerve fiber layer thickness and retinal ganglion cell complex in toxic and nutritional optic neuropathy

**DOI:** 10.1016/j.aopr.2022.100026

**Published:** 2022-01-25

**Authors:** Aishwarya Sriram, Yuan Miao, Prem Subramanian, Jeffery S. Schultz, Cheng Zhang

**Affiliations:** a Department of Ophthalmology, Montefiore Medical Center, Albert Einstein College of Medicine, Bronx, NY, USA; b Sue Anschutz Rodgers/UCHealth Eye Center, Department of Ophthalmology, University of Colorado School of Medicine, Aurora, CO, USA

## 1 Background

Optic neuropathy can result from ischemia, compression, inherited causes, as well as toxins or nutritional deficiencies. Toxic and nutritional optic neuropathy is defined as visual impairment from damage to the optic nerve due to medication or other ingested toxins, or from a nutritional deficiency. Clinically both tend to present as a painless bilateral progressive visual decline with dyschromatopsia and poor contrast sensitivity.^1^, ^2^, ^3^ The vision loss is typically a central or ceco-central scotoma due to high sensitivity of the papillomacular (PM) bundle to these insults, and the vision loss is usually not worse than 20/400 except in cases of methanol use. In early stages, the optic nerve may appear hyperemic or mildly edematous, however the optic nerve most commonly appears normal. It is generally believed with disease progression into late stages, optic atrophy becomes apparent.

Common etiologies for toxic optic neuropathy include various medications such as quinine, isoniazid, ethambutol, digitalis, amiodarone, vincristine, methotrexate, linezolid; abused substances such as tobacco, methanol, ethylene glycol; and heavy metals such as lead, mercury, and thallium. Nutritional deficiencies often include B-complex vitamins and folic acid, which may be further enhanced by alcohol abuse. Such toxins and deficiencies are thought to impair vascular supply, metabolism, and mitochondrial oxidative phosphorylation. This results in PM bundle damage and an associated decrease in retinal nerve fiber layer (RNFL) thickness on OCT,^1^^,^^3^ which can be used to detect disease before changes in the fundus appear^3^. In recent years, OCT-RGC complex analysis has become very important clinically in evaluating various optic neuropathies including glaucoma, a common optic nerve degenerative disease. Here we present five cases of toxic and nutritional optic neuropathy in which patients were found to have diffuse, early loss of the retinal ganglion cell (RGC) layer, with a relative preservation of the RNFL. Prior studies have shown that toxic or nutritional optic neuropathies can result in RGC loss,^4^ however our cases reveal that damage to the ganglion cell layer may occur first. Mitochondrial dysfunction, caspase activity, and neurotrophin deficiency have all been implicated in the death of ganglion cells,^5^, ^6^, ^7^ and certain toxins or nutritional deficiencies may affect these functions causing early ganglion cell death. This begs the question, *do some toxins or deficiencies have a propensity to damage the ganglion cell layer first? If so, why is the ganglion cell layer more predisposed to injury early in disease? Further, why does severe RGC loss persist for 1-*2 decades *with relatively normal RNFL thickness?*

## 2 Methods

A retrospective chart review was performed, reviewing patient cases of toxic or nutritional optic neuropathy. All patients were seen at one major academic center in the Bronx, NY. The review included collecting demographic and clinical information, including patient history and disease course. In addition, patients’ OCT and visual field testing was included in the review. Due to the retrospective nature of the review, the requirement of informed consents were waived.

## 3 Case presentation

### 3.1 Case 1

**Fig. 1:**
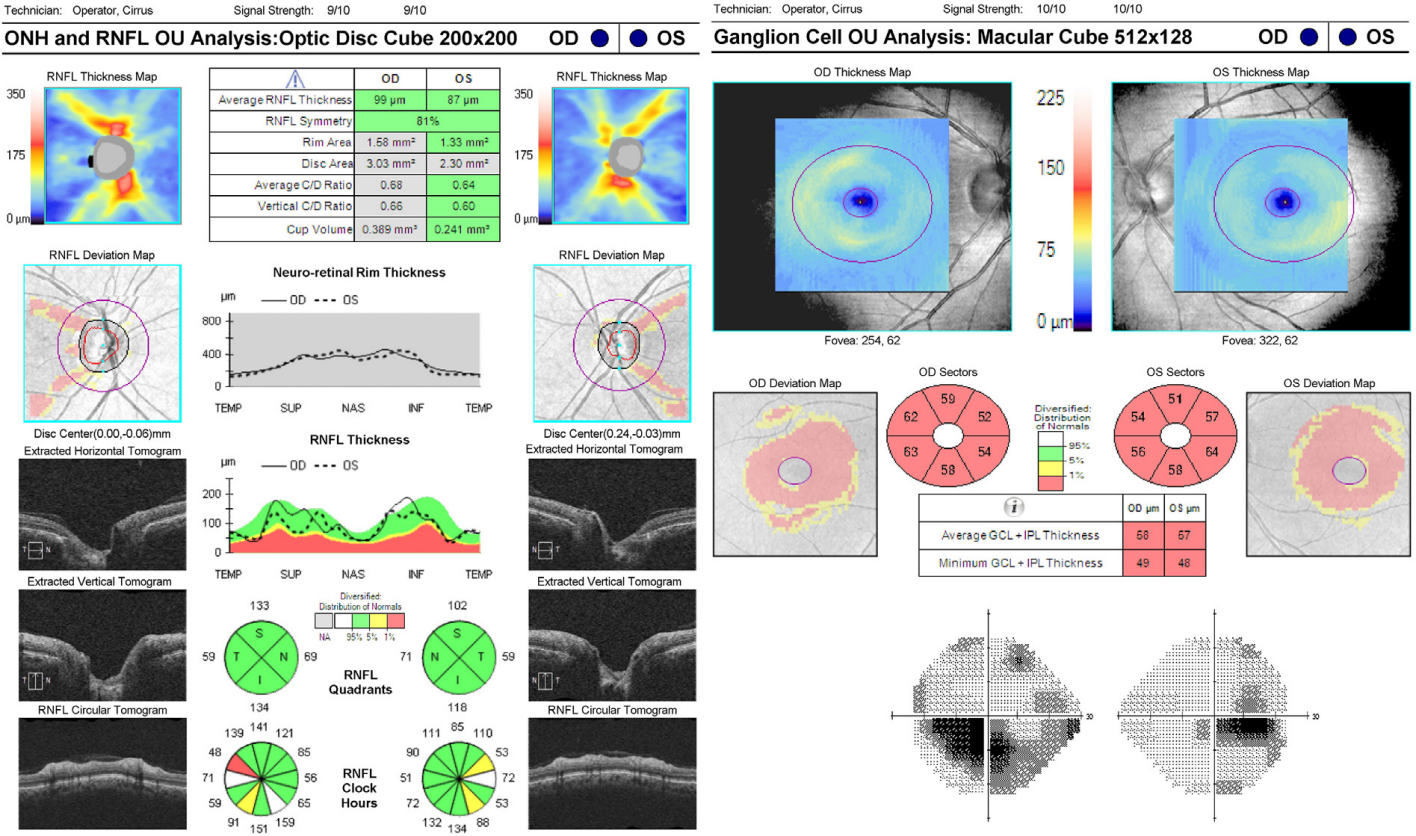
Case 1 RNFL, RGC, and Humphrey visual field RNFL: Retinal Nerve Fiber Layer; RGC: Retinal Ganglion Cell.

The first patient was found to have optic neuropathy from alcohol abuse, which is known to cause a nutritional optic neuropathy. This patient had moderate chronic alcohol use for over ten years, until January 2019 at which point the alcohol use disorder was in early remission. The patient also had a comorbidity of depression. On presentation, the patient complained of blurry vision for 1 year. Visual acuity was 20/70 in the right eye and 20/150 in the left eye. The right optic nerve showed mild pallor, and the left optic nerve was unremarkable. The patient had an extensive work up including MRI of the brain with and without contrast, and serum laboratory studies for vitamin levels. All workup was found to be non-contributory. On further testing, OCT-RNFL showed an average thickness of 99 in the right eye and 87 in the left eye. Both eyes only had mild focal thinning of the PM bundle in the temporal sector (Fig. 1). However, the OCT-macula showed diffuse loss of the ganglion cell layer. Average GCL ​+ ​IPL thickness was 58 in the right eye and 57 in the left eye. Visual field testing, consistent with optic neuropathy, demonstrated an inferior ceco-central scotoma of the right eye and an inferior ceco-arcuate scotoma of the left eye. The patient was then started on a multi-vitamin, B12, and thiamine. The standard protocol consisted of intramuscular injections for the first three months followed by oral maintenance dosing, and the patient will be monitored in our clinic.

### 3.2 Case 2

**Fig. 2:**
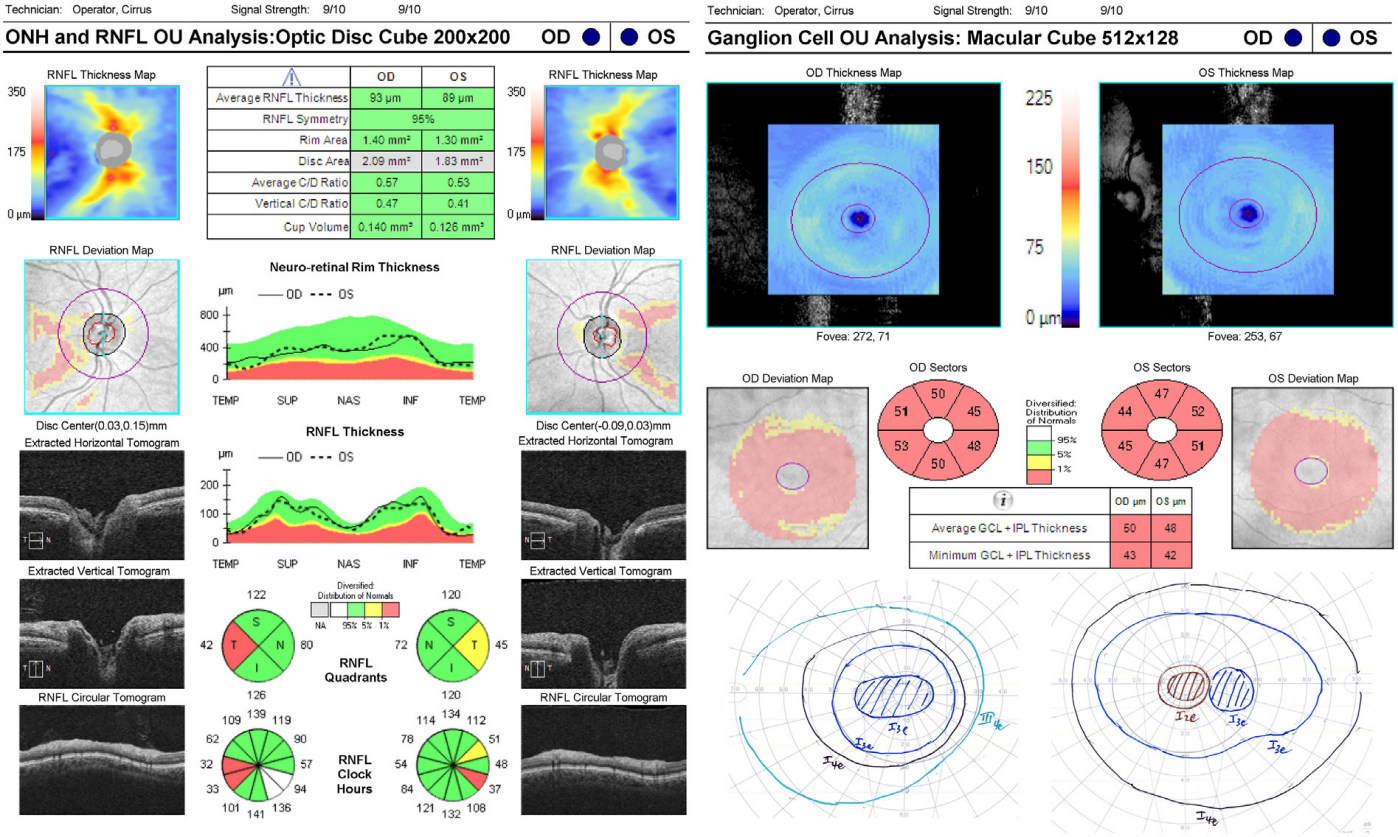
Case 2 RNFL, RGC, and Goldmann visual field RNFL: Retinal Nerve Fiber Layer; RGC: Retinal Ganglion Cell.

Case 2 is another case of alcohol induced optic neuropathy associated with nutritional deficiency. The patient was a 38 year old female who had a history of alcohol abuse with associated pancreatitis. The patient also had a recent diet change in which she stopped eating meat, but continued eating seafood. She presented with worsening weight loss and blurry vision. CT head and serum laboratory testing, including serum evaluation for vitamin deficiencies, were performed at an outside hospital and reported to be normal. On presentation, her visual acuity was 20/70 in both eyes, fundus exam showed mild temporal pallor of the optic nerves in both eyes, and color vision was 1/11 Ishihara plates in both eyes. Similar to the aforementioned patient, this patient had mild focal thinning of the PM bundle temporally with normal average RNFL thickness (93 in the right eye and 89 in the left eye) (Fig. 2). However, there was diffuse thinning of the ganglion cell layer with an average GCL ​+ ​IPL thickness of 50 in the right eye and 48 in the left eye. As expected, the patient had a central scotoma of the right eye and a ceco-central scotoma of the left eye as seen on Goldmann visual field testing. This patient was started on multivitamins, after which her vision improved to 20/40 in both eyes and her color vision improved to 6/11 Ishihara plates in both eyes.

### 3.3 Case 3

**Fig. 3:**
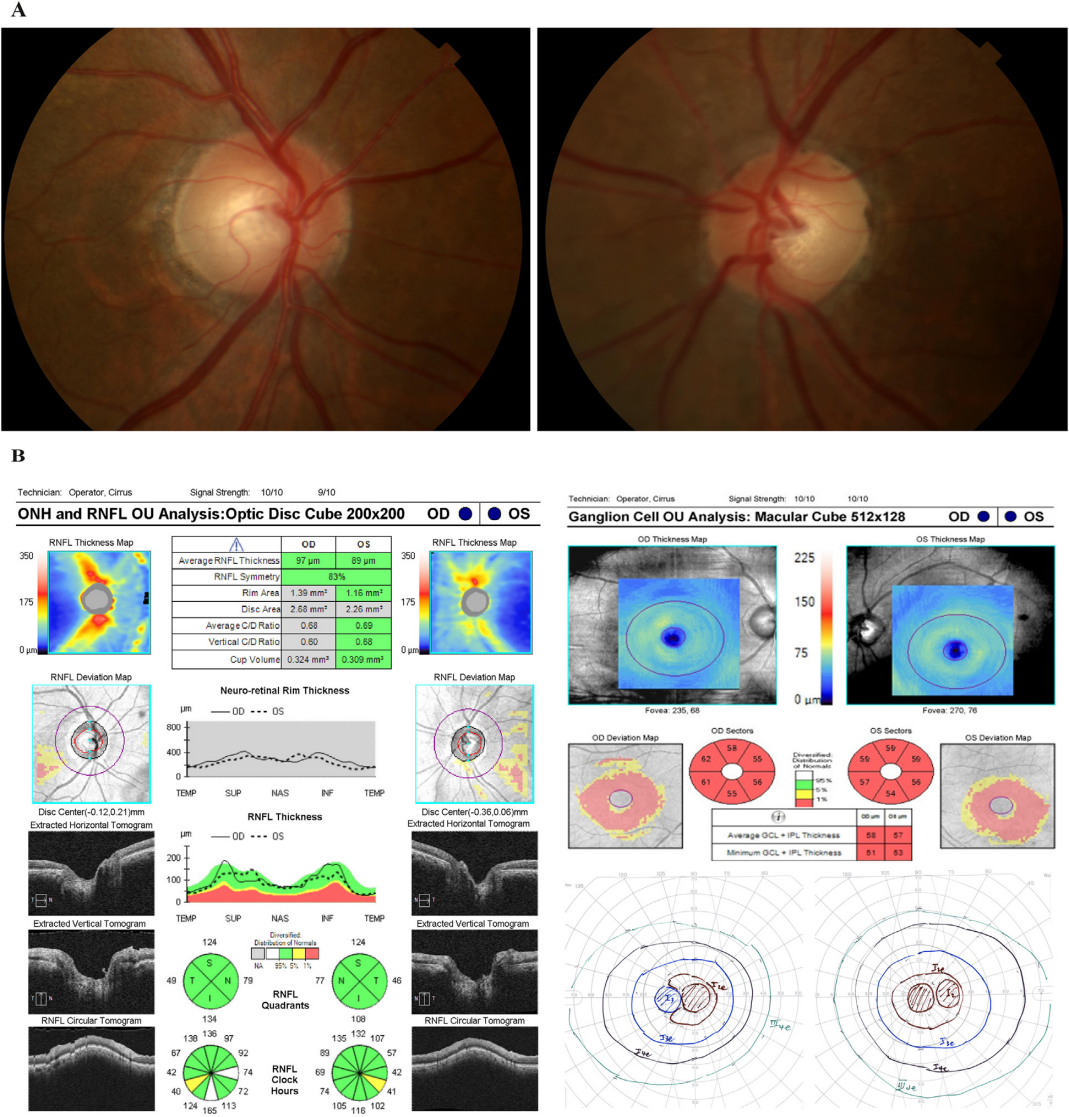
(A) Case 3 Optic nerve photos. (B) RNFL, RGC, and Goldmann visual field. RNFL: Retinal Nerve Fiber Layer; RGC: Retinal Ganglion Cell.

The third case is that of a 60 year old male with chronic tobacco use. The patient had an extensive smoking history, with over 20 years of tobacco use, and he presented with chronic vision loss for the last 10 years. Extensive workup including MRI, serum laboratory testing, and genetic testing was found to be negative. On presentation, his vision was 20/200 in both eyes. Color vision was preserved with 10/11 on Ishihara plates identified with a magnifying lens. Optic nerves had minimal pallor OU (Fig. 3A). The patient also had ERG, VEP, and mfERG for further evaluation, which were found to be normal. Testing showed an intact RNFL with no notable thinning, with an average thickness of 97 in the right eye and 89 in the left eye (Fig. 3B). However the ganglion cell layer showed diffuse thinning of both eyes with an average GCL ​+ ​IPL thickness of 58 in the right and 57 in the left eye. Goldmann visual field testing consistently showed a central or ceco-central scotoma in eyes.

### 3.4 Case 4

**Fig. 4:**
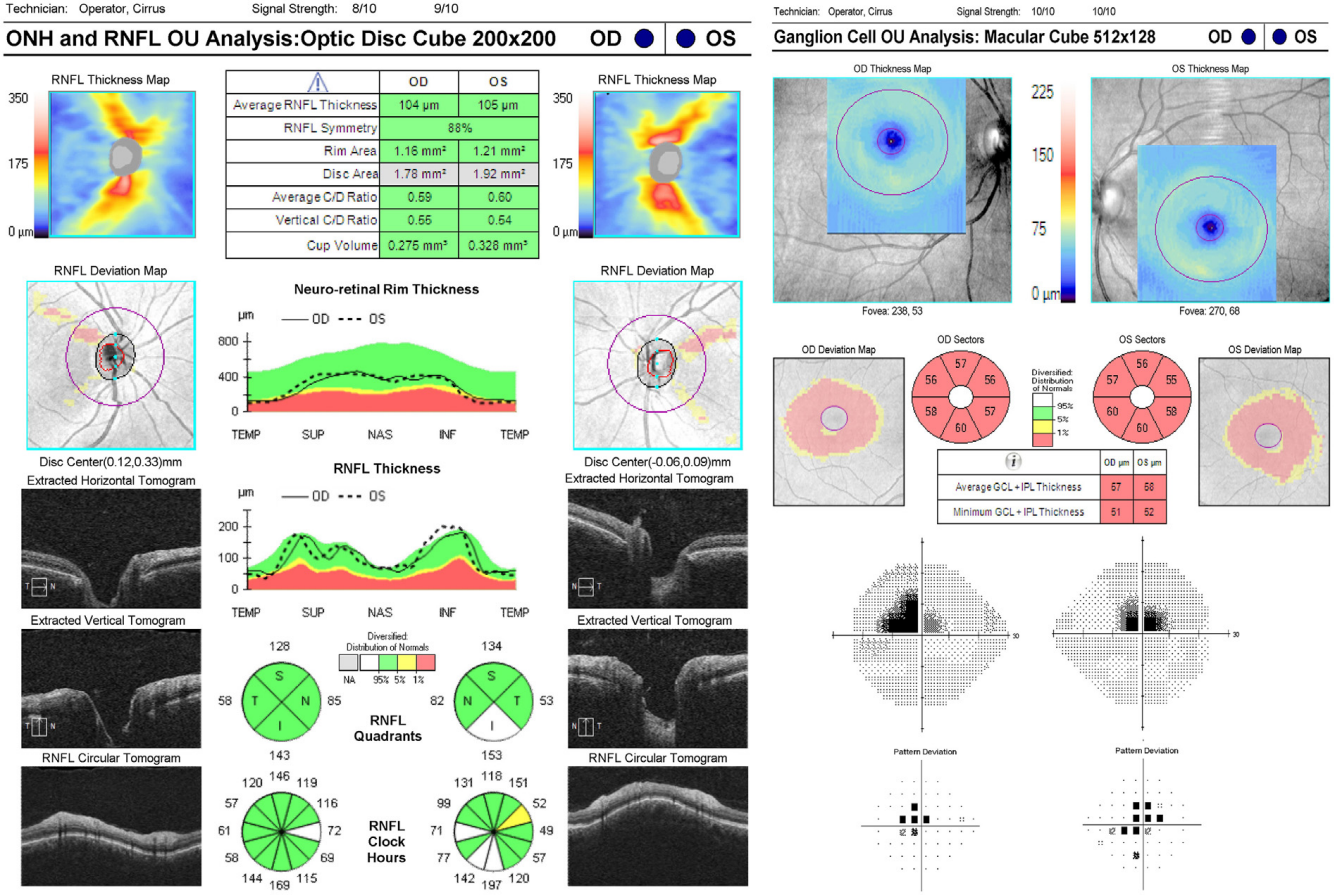
Case 4 RNFL, RGC, and Humphrey visual Field RNFL: Retinal Nerve Fiber Layer; RGC: Retinal Ganglion Cell.

The fourth case is another case of toxic optic neuropathy from tobacco use with a possible nutritional deficiency component. The patient had smoked marijuana mixed with raw tobacco leaves daily for the last 20 years. The patient did not smoke cigarettes or cigars, and had minimal alcohol use. Of note, the patient had also been a vegetarian for the last 20 years. The patient presented with complaints of blurred vision for the past month in both eyes, with progressive worsening. Visual acuity was 20/200 on presentation in both eyes and the patient also demonstrated decreased color vision in both eyes. Additional workup including laboratory evaluation for vitamin deficiency as well as CT scans of both the head and orbits were negative. OCT testing revealed a preservation of the nerve fiber layer with an average thickness of 104 in the right eye and 105 in the left eye (Fig. 4). However the ganglion cell layer was diffusely thinned with an average GCL ​+ ​IPL thickness of 57 in the right eye and 58 in the left eye. Visual field testing showed central scotomas in both eyes which were worse in the superior visual field.

### 3.5 Case 5

**Fig. 5:**
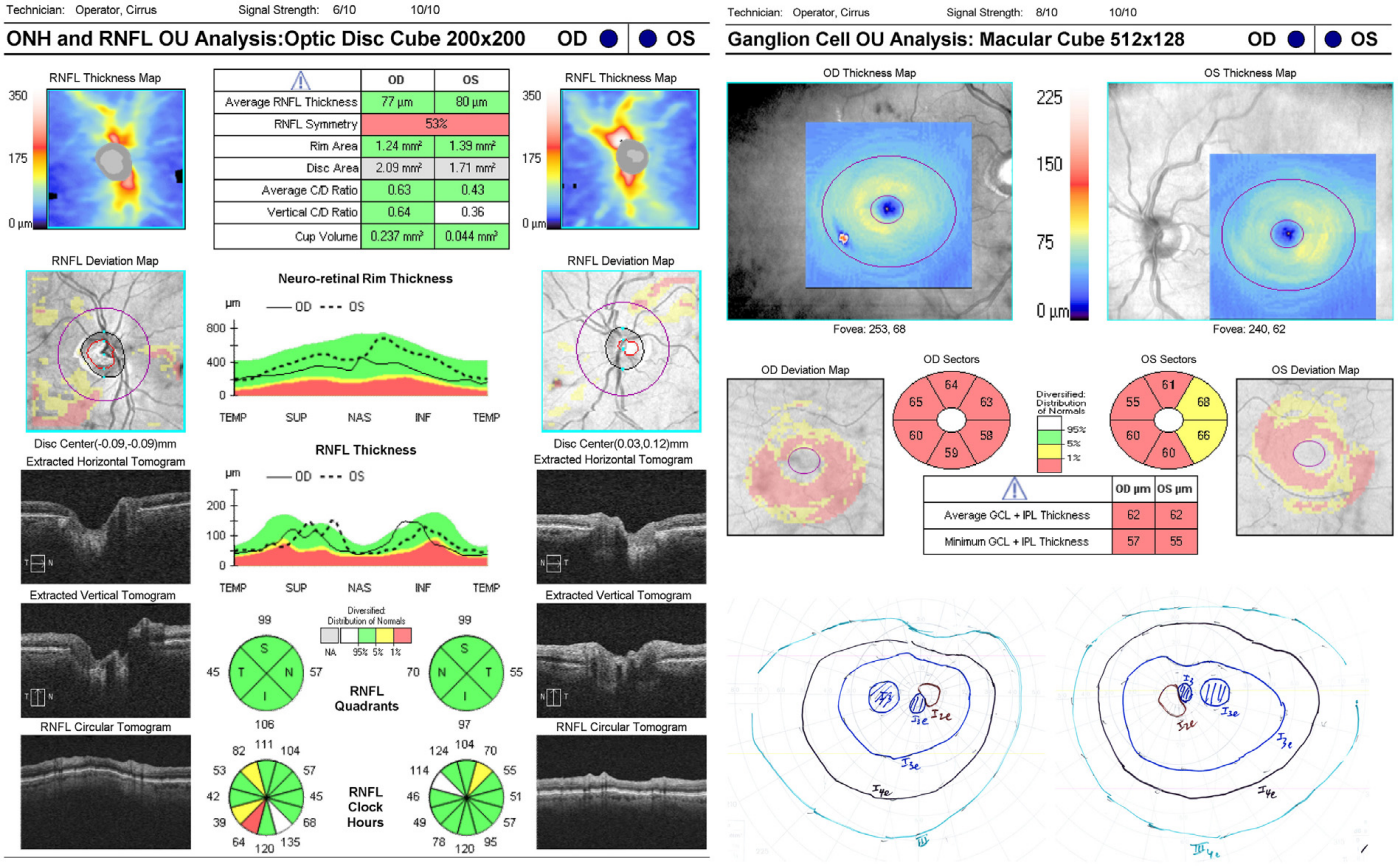
Case 5 RNFL, RGC and Goldmann visual field RNFL: Retinal Nerve Fiber Layer; RGC: Retinal Ganglion Cell.

The fifth case is that of a patient with optic neuropathy secondary to ethambutol use. The patient is a 77-year old male who had been treated with ethambutol 15 mg/kg per day for the past 7 months for an atypical lung infection. He presented with gradual visual decline which began in the left eye followed by the right eye. His visual acuity on presentation was 20/300 in the right eye and count fingers in the left eye. An MRI brain was reported normal. Serum evaluation for vitamin deficiencies were ordered but never completed. The patient underwent OCT and visual field testing, and his results were consistent with all of the aforementioned patients. RNFL average thickness was 77 in the right eye and 80 in the left eye, with mild focal thinning (Fig. 5). The ganglion cell layer was diffusely damaged in the right eye with an average thickness of 62, and had more pronounced damage nasally in the left eye with an average thickness of 62. Central scotomas were identified in both eyes on Goldmann visual field testing.

### 3.6 Case 6

**Fig. 6:**
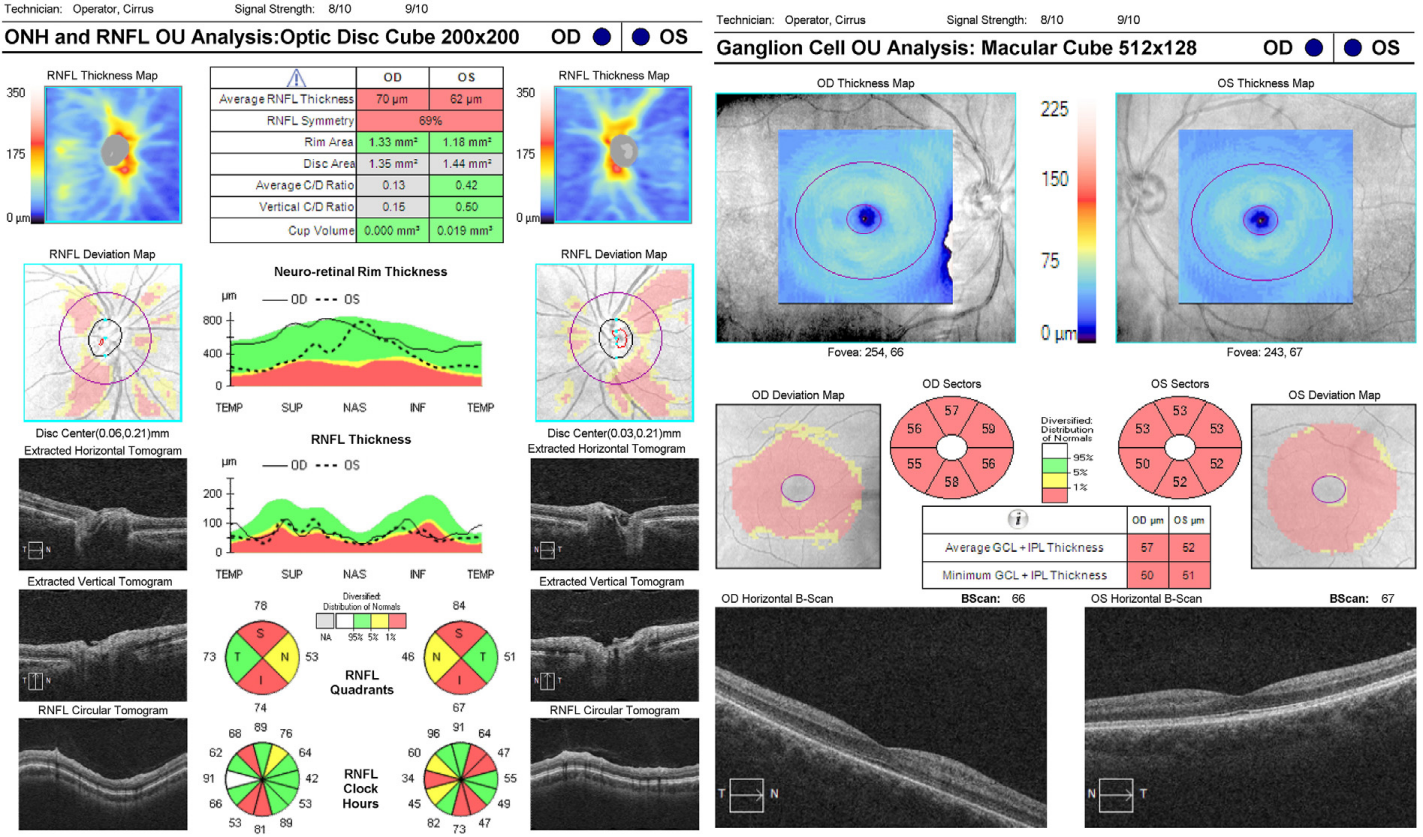
Case 6 RNFL and RGC RNFL: Retinal Nerve Fiber Layer; RGC: Retinal Ganglion Cell

The sixth case is that of a patient with dominant optic atrophy, presented for comparison. This patient had positive genetic testing for dominant optic atrophy. The patient had a ceco-central scotoma in both eyes as expected (not shown). However, the OCT, which was done early in the disease course, revealed moderate to severe damage to both the nerve fiber layer and ganglion cell layer (Fig. 6). This damage to both the nerve fiber layer and ganglion cell layer early in dominant optic atrophy is notably different from the ganglion cell damage with preservation of the nerve fiber layer we have presented in the aforementioned cases.

## 4 Conclusions

We have presented here five cases of toxic and nutritional optic neuropathy in patients with diffuse loss of the ganglion cell layer. The patients all presented with subjective visual decline and had a history of alcohol abuse, tobacco use, or ethambutol exposure with or without a nutritional deficiency. We also published 2 cases of toxic optic neuropathy due to cassava consumption in a couple in 2018.^8^ Similar to those two patients, the patients presented here were all found to have a decline in visual acuity and color vision, with central or ceco-central scotomas. Most notably however, all patients demonstrated diffuse loss of the retinal ganglion cell complex with only minimal loss of the nerve fiber layer. The cases described indicate that toxic substances or nutritional deficiencies may have a propensity to affect the ganglion cell body diffusely before the nerve fiber layer is affected.

RGC layer loss has been demonstrated in ischemic optic neuropathy, compressive optic neuropathy, and inherited optic neuropathies including Leber's hereditary optic neuropathy and dominant optic atrophy.^9^, ^10^, ^11^ However, the primary defect of these optic nerve diseases is at the level of the optic nerve axons, with secondary death of the ganglion cell body. For example, dominant optic atrophy has been shown to cause thinning of both the nerve fiber layer and ganglion cell layer early in disease,^12^ and is seen in case six. However, to our knowledge there has been no described preferential loss of either layer. In cases of optic neuritis or non-arteritic ischemic optic neuropathy, damage to axons results in ganglion cell body death within days to weeks while RNFL thinning occurs in 1–2 months.^13^, ^14^, ^15^, ^16^

The cases in our series indicate that toxic and nutritional optic neuropathies also cause thinning of the RGC layer, yet contrastingly, there is relative sparing of RNFL. Prior studies have indeed shown the effect of toxins or nutritional deficiencies on retinal ganglion cells. Ethambutol, for instance, has been found to be toxic to ganglion cells both in vivo and in vitro. Studies have shown that it can affect both retinal neurons and ganglion cell body axons. Ethambutol requires endogenous glutamate in order to exert its toxic effects, and glutamate in and of itself has also been found to be toxic to both ganglion cell bodies and their axons.^2^^,^^17^ However, Heng et al.^2^ found that ethambutol resulted in loss of the retinal ganglion cell layer, without affecting other layers of the retina. This indicates that the toxic effects of ethambutol may be seen in the cell bodies first, with a relative preservation of the nerve fiber layer initially. Additionally, a study by Vieira et al. found that there was a significant decrease in retinal ganglion cell layer thickness and volume in patients with toxic/nutritional optic neuropathies from ethambutol, alcohol, or tobacco. The decrease in thickness and volume was greater with greater time of disease.^4^ This study, however, did not assess the nerve fiber layer thickness in these patients.

Retinal ganglion cell death is thought to occur due to mitochondrial dysfunction, as these cells are particularly vulnerable given their high energy demand, long axons, and complex dendritic arbors.^6^ As such, there may be certain substances common to the toxins described above that interfere with mitochondrial function and infer a particular risk for optic neuropathy. Caspases are also known to play a role in ganglion cell autophagy,^5^ and perhaps be upregulated by certain toxins or deficiencies. A lack of neurotrophins is also known to cause ganglion cell death, with factors such as ischemia impairing vascular supply and contributing to such death.^7^ Thus it is also possible that certain toxins impair adequate neurotrophin delivery to ganglion cell bodies, causing cell death in a similar way.

In the cases we have described, however, not only did the patients have diffuse ganglion cell layer degeneration, but they had relative preservation of the nerve fiber layer. The mechanism for differential ganglion cell death before nerve fiber layer death is unclear. Ganglion cells may be more susceptible to injury early on in the disease process. They may have differing characteristics from the nerve fiber layer which make them susceptible to degeneration from toxins or nutritional deficiencies. Perhaps certain substances are prone to affect neuronal cell bodies as opposed to axons, causing ganglion cell layer death first. In inherited optic neuropathies, which are related to mitochondrial disease, the nerve fiber layer is damaged first because the axons contain abundant mitochondria and PM bundle nerve axons are highly energy dependent and are thus most vulnerable. As such, in mitochondrial diseases, the very early complication for the heart is conduction block due to nerve axonal damage.

While the mechanism for early and preferential ganglion cell death is unclear, we surmise that ganglion cells may have predisposing factors making them prone to death before the nerve fiber layer. With further investigation, RGC layer analysis with toxic or nutritional optic neuropathy may prove useful in identifying severity of disease or monitoring for progression. Ganglion cell layer analysis may be helpful in identifying disease early on, particularly as the loss of ganglion cells with preservation of nerve fiber layer is seen early on OCT while optic atrophy and pallor may not be evident until the end stages of disease. Utilization of retinal ganglion cell layer analysis may also prove useful in managing the treatment of these optic neuropathies, and additional laboratory investigation could provide invaluable insight into the early ganglion cell body toxicity. The cases described here indicate that there is likely a significant relationship between ganglion cell death and toxic/nutritional optic neuropathy. As such, further investigation into this topic and the mechanism for preferential ganglion cell death would be of tremendous value.

## Study Approval

The authors confirm that any aspect of the work covered in this manuscript that involved human patients or animals was conducted with the ethical approval of all relevant bodies and the study was performed in accordance with the Declaration of Helsinki.

## Author Contributions

The authors confirm contribution to the paper as follows: Conception and design of study: CZ, PS, JSS; Data collection: CZ, AS; Analysis and interpretation of results: CZ, PS; Drafting the manuscript: CZ, AS, YM; All authors reviewed the results and approved the final version of the manuscript.

## Acknowledgments

Thanks to all the peer reviewers for their opinions and suggestions.

## Funding

This research did not receive any specific grant from funding agencies in the public, commercial, or not-for-profit sectors.

## Conflict of Interest

The authors declare that they have no known competing financial interests or personal relationships that could have appeared to influence the work reported in this paper.
